# Up-regulated GRB7 protein in gastric cancer cells correlates with clinical properties and increases proliferation and stem cell properties

**DOI:** 10.3389/fonc.2022.1054976

**Published:** 2023-01-04

**Authors:** Yuan-yuan Pei, Jian Ran, Lijuan Wen, Xiaoyi Liu, Li Xiang, Weiqiang Liu, Fengxiang Wei

**Affiliations:** ^1^ The Central Laboratory, Longgang District Maternity & Child Healthcare Hospital of Shenzhen City, Shenzhen, China; ^2^ The Digestive Department, Longgang District People’s Hospital of Shenzhen City, Shenzhen, China

**Keywords:** GRB7 adaptor protein, gastric cancer, proliferation, stem cell, clinical properties

## Abstract

**Introduction:**

It has been reported that GRB7 is closely related to a variety of human solid tumors, but its role in gastric cancer has not been reported yet. The purpose of this study was to investigate the expression level and intracellular effects of GRB7 in human gastric cancer.

**Methods:**

Real-time fluorescent quantitative PCR and Western blot were used to detect the expression of GRB7 in gastric cancer cell lines. The immunohistochemical staining and SPSS analysis verified the GRB7 protein expression. Stable gastric cancer cell lines, MTT experiments, clone formation experiments, cell cycle flow cytometry experiments, sphere formation experiments and lateral subpopulation cell sorting experiments were conducted to investigate the role of GRB7 in gastric cancer cells.

**Results:**

We found that the expression of GRB7 in gastric cancer cell lines was higher than that of the corresponding normal gastric epithelial cells, and correspondingly higher in gastric cancer tissues than its paired adjacent tissues. GRB7 protein was expressed more highly in cancer tissues than in adjacent tissues. GRB7 protein expression levels were positively correlated with the clinical stage of gastric cancer patients, and negatively correlated with the survival prognosis of patients. GSEA analysis of GRB7 mRNA levels in gastric cancer tissues and normal gastric epithelial tissues from public databases showed that GRB7 may affect cell proliferation and related processes of intracellular stem cells. GRB7 can promote the proliferation of gastric cancer cells and is positively related to the self-renewal ability of gastric cancer stem cells.

**Discussion:**

This study shows that GRB7 molecules highly expressed in gastric cancer tissues can promote the proliferation of gastric cancer cells and increase the proportion of gastric cancer stem cells, so it is expected to become a diagnostic molecule or potential therapeutic target for gastric cancer.

## Introduction

Worldwide, gastric cancer has become the fourth most common cancer due to its high incidence, and it is also the second leading cause of cancer-related deaths (1). Epidemiological studies have shown that the incidence of gastric cancer in adolescents is gradually increasing, and many patients with gastric cancer are already at an advanced stage when they are diagnosed (2). Traditional gastric cancer treatment strategies, including surgery, chemotherapy and radiotherapy, are still not ideal in improving advanced gastric cancer (3). However, molecular targeted therapy has shown its unique curative effect in the treatment of gastric cancer, especially advanced gastric cancer. Molecular targeted therapy is a drug designed for oncoprotein molecules or oncogene fragments that specifically binds to cancer-causing sites in the body, so as to cause tumor cell death without affecting normal tissue cells around the tumor (4). The molecular targets of gastric cancer that have been studied in depth mainly include EGFR, HER-2, VEGF, VEGFR, mTOc-MET, HGF, etc (3, 5, 6). The anti-EGFR monoclonal antibody Cetuximab was found in a phase II randomized trial to median survival (mOS) of patients with metastatic gastric cancer (6); the application of the anti-HER-2 monoclonal antibody Trastuzumab can make HER-2 patients with positive advanced gastric cancer benefit and have been approved for the first-line treatment of advanced gastric cancer (7); Ramucirumab targeting VEGFR2 has a good anti-tumor effect (8); multi-target tyrosine kinase inhibitor Regorafenib as a multi-target phosphokinase inhibitors have multiple anti-tumor effects (9). In summary, deepening the research and understanding of the molecular pathogenesis of gastric cancer will be beneficial to the early diagnosis and targeted therapy of gastric cancer.

As a typical adaptor protein, GRB7 consists of a pro-rich region at the N-terminal, RA (ras-associated) region, PH (pleckstrin homology) region, BPS (between pleckstrin and src) region, and C-terminal SH2 (src same as src Source 2) regional (10). Studies have shown that these domains of GRB7 have the ability to interact with a variety of receptor tyrosine kinases and their downstream partners to mediate multiple cell signal transduction pathways, such as ERK, Ras, and Akt (11, 12). The relationship between GRB7 expression and cancer development is worth exploring. In terms of molecular expression level, GRB7 has been found to be overexpressed in a variety of human cancer tissues, including bladder cancer, breast cancer, ovarian cancer and hepatocellular carcinoma (13–16). At the same time, the GRB7 protein expression level has been deeply analyzed and it is often negatively correlated with the patient’s prognosis, that is, the higher the GRB7 expression, the worse the patient’s prognosis (13). In terms of molecular function, GRB7 participates in the regulation of multiple signal transduction pathways in the physiological and pathological processes involved in many aspects of tumorigenesis and development (17). GRB7 has been found to have an important growth promoting function in human breast cancer cell lines, and inhibition of GRB7 will reduce the motility of breast cancer cells and promote cell death (18, 19). Overexpression of GRB7 in cervical cancer has been found to promote cervical cancer cell invasion and inhibit cell apoptosis, while knockdown of GRB7 in esophageal adenocarcinoma reduces proliferation and induces apoptosis (20, 21). It can be seen that a variety of tumor models have verified the important role of GRB7 molecules in the malignant process of tumors. However, the expression of GRB7 protein and its function in in human gastric cancer cells have not been reported yet.

Here, we report for the first time the over-expression of GRB7 mRNA and protein expression levels in gastric cancer and its negative correlation with patient prognosis. In addition, GSEA analysis of GRB7 mRNA levels in gastric cancer tissues and normal gastric epithelial tissues in public databases predicted its possible molecular functions. Furthermore, we established a stable cell line and verified the molecular function of GRB7 in gastric cancer cells. In conclusion, our current research shows that the GRB7 molecules highly expressed in gastric cancer tissues can promote the proliferation of gastric cancer cells and increase the proportion of gastric cancer stem cells.

## Materials and methods

### Cell lines and tissues

Serum-free medium (Invitrogen Life Technologies, Carlsbad, CA, USA) supplemented with epithelial growth factor, bovine pituitary extract and antibiotics was used to culture normal gastric epithelial cells (NGEC1 and NGEC2). Dulbecco’s Modified Eagle Medium (DMEM; Gibco, Rockville, MD, USA) supplemented with 10% fetal bovine serum was used to cultivate 6 gastric cancer cell lines (HGC27, KATO3, NCI-N87, AGS, MKN-45, MGC-803). The cells were briefly digested with 0.25% trypsin (Gibco-Invitrogen, USA). The main components of cell culture freezing medium are high-sugar DMEM, 20% high-quality fetal bovine serum and 10% DMSO. The fresh tissues of 8 gastric cancer patients had been confirmed by paraffin embedding, sectioning and hematoxylin-eosin staining.

### Real-time quantitative PCR, Western blot and the establishment of stable cell lines

The experimental procedures of Real-time quantitative PCR (qPCR), Western blot and the establishment of stable cell lines were performed in accordance with previous studies (22). The primers and antibodies used in this study have been listed in our published articles (13).

### Immunohistochemistry and statistical analysis

In this study, we used immunocytochemistry to detect GRB7 in 210 cases of gastric cancer. The working concentration of anti-GRB7 was 1:200 (1:200, Santa Cruz Biotechnology, USA). Each slice was photographed as a whole, and analyzed by the axiovision rel.4.6 computer image analysis system and automatic measurement program (Carl Zeiss, Oberkochen, Germany), and the average optical density (MOD) of the slice was obtained and calculated. All specimens were divided into GRB7 high expression group and GRB7 low expression group according to MOD median. Chi-square test was used for comparison between groups. Two-tailed P value less than 0.05 was statistically significant. The tumor genome map (TCGA) used in this study included 37 normal gastric tissues and 409 gastric cancer tissues. Among them, 33 pairs of data are paired data from the same patient. Gene set enrichment analysis (GSEA) was used to evaluate the correlation between GRB7 gene and cancer phenotypes.

### 3-(4,5-Dimethyl-2-thia-zolyl)-2,5-diphenyl-2-H-tetrazolium bromide (MTT) assay

The logarithmic phase cells were digested with trypsin and centrifuged after termination with complete medium. The cell count was adjusted to 5×10^4^/ml. 100 UL of the cell suspension was added to 96-well plate (5000 cells/well), and then put into the incubator for culture. After 48 hours, 10ul MTT solution (5mg/ml; Santa Cruz Biotechnology, Inc., Dallas, TX, USA) was added to each well for further culture. After 4 hours, the supernatant of the fully formed crystals was carefully removed. 150ul dimethyl sulphoxide (DMSO; Sigma-Aldrich)) was added to each well. After 10 minutes of low-speed oscillation, the crystal solution was placed on the enzyme-linked immunosorbent assay (optical density, OD 490nm) to measure the absorbance of each well.

### Colony formation assay

Cells in log phase were trypsinized with 0.25% trypsin and dispersed into single cells by gentle pipetting. 1000 cells were inoculated into a six well plate with 10ml complete medium in each well, and the cells were dispersed evenly by gently rotating. The cells were cultured in the incubator for 2-3 weeks. When the cells were washed with 5 ml of formalin for 4 minutes, the cells could be seen by naked eyes. Then remove the fixing solution, add appropriate amount of nitro blue tetrazolium (Sigma) for 10-30 minutes, then wash the staining solution slowly with running water and dry it with air. The plate was inverted and a piece of transparent film with grid was superimposed. The number of clones with more than 10 cells was counted by microscope (low power microscope) and the clone formation rate was calculated. Clone formation rate = (clones/inoculated cells) × 100%.

### Cell cycle analysis by flow cytometry

Cells in logarithmic growth phase were harvested and centrifuged at 800 rpm for 5 minutes. The cell precipitates were washed twice with pre-cooled PBS and pre-cooled with 75% ethanol, then placed in 4°C refrigerators. After 4 hours, the cell precipitate was washed once with 3ml PBS, then 400ul ethidium bromide (EB; Sigma; 50ug/ml); and 100ul RNase A (100 ug/ml) were added. The cells were incubated at 4°C for 30 min, and then detected by flow cytometry according to the standard procedure (30,000 cells were counted). Results the cell cycle was analyzed by ModFit software.

### Spheroid culture

The logarithmic phase cells were centrifuged and washed twice with PBS; the cells were resuspended and counted in dulbecco’s modified eagle medium(DMEM; Gibco) supplemented with B27 supplement (Gibco), 20ng/ml basic fibroblast growth factor(bFGF;Sigma) and 20ng/ml epidermal growth factor (EGF; Sigma). 1000 cells were added into each well of the low adsorption six-well plate and 4ml of culture medium was added; after 10 days, the spheroidization was observed, photographed and counted.

### Side population cell sorting

The cells in logarithmic phase were digested and counted, and the concentration of cells was adjusted to 1×10^6^/ml in DMEM medium containing 0.1% fetal bovine serum (FBS; Gibco). Each cell was divided into test group and control group. Hoechst 33342 (6μg/ml; Sigma) was added to both groups, and Verapamil (50μmol/ml; Sigma, St Louis, USA) was added to the control group. After 90 min in 37°C dark water bath, the cells were placed on ice for 10min to stop staining. The cell concentration was adjusted to 1×10^7^/ml with ice pre-cooled DMEM and 0.15 mg/ml DNase I (Sigma) was added; Propidium iodide (PI; 2μg/ml) was added before cell operation. The cells filtered by 400 filter were detected by flow cytometry. The blue and red parts of the scattered light were detected by 450nm and 675nm edge long pass filters, respectively. Using Hoechst red as the x-axis and Hoechst blue as the y-axis, the two-dimensional scatter plot was made. The areas with low Hoechst red and low Hoechst blue and basically missing in verapamil group were set as the “gate” of SP cells. The percentage of cells in the “gate” represents the proportion of SP cells.

## Results

### GRB7 is up-regulated in gastric cancer cells and tissues

**Figure 1:**
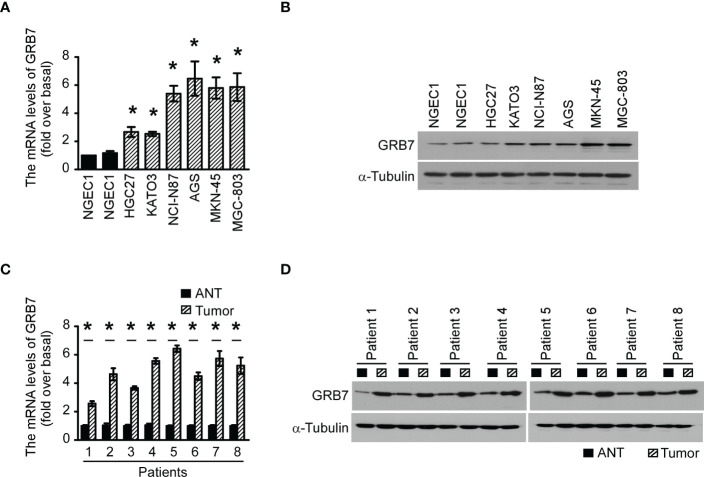
The expression of GRB7 in gastric cancer cells and gastric cancer tissues was up-regulated. **(A)** The qPCR results showed that GRB7 levels in the 6 human gastric cancer cell lines were significantly higher than the two normal gastric epithelial cells (NGEC1 and NGEC2). The statistics were compared with NGEC1. *P<0.05. **(B)** Western blot analysis showed that GRB7 protein levels were correspondingly increased in 6 human gastric cancer cells. **(C)** The GRB7 mRNA level in gastric tumor tissues of 8 patients was significantly higher than that of their paired adjacent tissues. **(D)** The amount of GRB7 protein in matched gastric cancer tissues was also higher than that in their respective paired adjacent tissues. The results of each tumor were statistically compared with the adjacent normal tissue data. *P<0.05. Gastric tumor tissue is abbreviated as tumor, and adjacent normal tissue is abbreviated as ANT.

Real-time quantitative PCR results revealed that GRB7 levels in the 6 human gastric cancer cell lines were significantly higher than the two normal gastric epithelial cells (NGEC1 and NGEC2, **Figure 1A**). Western blot analysis showed that GRB7 protein levels were correspondingly increased in 6 human gastric cancer cells (**Figure 1B**). In order to verify the results detected in the cell lines, we collected 8 pairs of gastric cancer/non-cancer specimens after surgery. As shown in the **Figures 1C, D**, the mRNA level and protein level in matched gastric cancer tissues(tumor) was also higher than that in their respective paired adjacent tissues(ANT). Take together, the expression of GRB7 is up-regulated in gastric cancer cells and gastric cancer tissues.

### Immunohistochemistry reveals the close relationship between GRB7 and clinical features of gastric cancer

**Figure 2:**
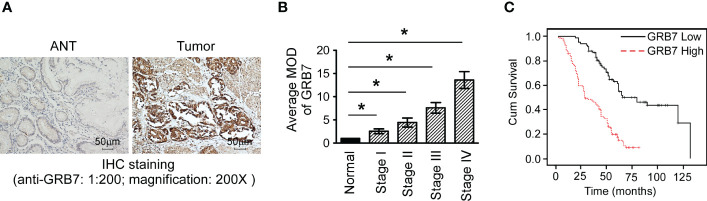
The expression of GRB7 protein in immunohistochemistry was related to the clinical features of gastric cancer. **(A)** Typical photos of a paraffin section showed that the expression of GRB7 protein in cancer tissues was significantly higher than that in normal gastric tissues. **(B)** Statistical quantification of mean optical density (MOD) based on different clinical grades of gastric cancer showed that GRB7 protein expression increases with the clinical stage. The value of each stage was compared with the mean value of GRB7 staining of non-cancerous tissues. *P<0.05. **(C)** Kaplan-Meier curve showed that the prognosis of gastric cancer patients in the GRB7 high expression group was worse.

We used immunohistochemical methods to verify and analyze the expression characteristics of GRB7 protein and its relationship with clinical diagnosis and prognosis. The brown color representing GRB7 protein is widely distributed and black in gastric cancer lesions. In contrast, the brown distribution in non-cancerous gastric mucosal tissue is limited and lighter in color. It can be seen that GRB7 is mainly located in the cell membrane in normal gastric mucosal tissues, while in malignant cells it is mainly located in the cytoplasm and nucleus(**Figure 2A**). Statistical quantification of mean optical density (MOD) based on different clinical grades of gastric cancer showed that GRB7 protein expression increases with the clinical stage (**Figure 2B**). Further statistical analysis showed that the expression of GRB7 was not related to the patient’s age, but was closely related to the clinical stage and pathological grading of gastric cancer. In detail, GRB7 was positively correlated with tumor growth size (T classification), lymph node metastasis (N classification), and distant organ and tissue metastasis (M classification) (**Table 1**). Multivariate analysis of Cox proportional hazards model showed that the expression of GRB7 was an independent prognostic factor of gastric cancer as well as T classification, N classification, M classification and pathological differentiation (**Table 2**). Kaplan-Meier curve showed that the prognosis of gastric cancer patients in the GRB7 high expression group was worse (**Figure 2C**). Take together, the expression level of GRB7 protein is positively correlated with the higher clinical grade of gastric cancer and the worse prognosis of patients.

**Table 1:** Correlation between GRB7 expression and clinicopathologic characteristics of gastric cancer patients.

Characteristics	GRB7		
	Low or none	High	P value
Age(y)			
≥60	38 (18.1%)	69 (32.9%)	0.142
<60	45 (21.4%)	58 (27.6%)	
Gender			
Male	80(38.1%)	69 (47.1%)	<0.001
Female	45 (1.4%)	58 (13.3%)	
Clinical stage			
I	16(7.6%)	2 (1.0%)	<0.001
II	33 (15.7%)	17 (8.1%)	
III	31(14.8%)	63 (30.0%)	
IV	3 (1.4%)	45 (21.4%)	
T classification			
T1	14(6.7%)	3 (1.4%)	<0.001
T2	34 (16.2%)	17 (8.1%)	
T3	9(14.8%)	20 (30.0%)	
T4	83(1.4%)	127 (21.4%)	
N classification			
N0	36(17.1%)	15 (7.1%)	<0.001
N1	22 (10.5%)	41 (19.5%)	
N2	25(11.9%)	63 (30.0%)	
N3	0(0.0%)	8 (3.8%)	
Metastasis			
No	80(38.1%)	82 (39.0%)	<0.001
Yes	3 (1.4%)	45 (21.4%)	
Pathologic differentiation			
No (undifferentiated)	0 (0.0%)	5 (2.4%)	<0.001
Yes (differentiated)	83 (39.5%)	122 (58.1%)	

**Table 2:** Multivariate analyses of various prognostic parameters in patientswith gastric cancer Cox-regression analysis.

	B	Regression coefficient (SE)	Wald interval	Sig.	Exp(B)	95% Cl for Exp(B)	
						Lower	Upper
**GRB7**	0.664	0.211	9.889	0.002	1.943	1.284	2.939
**T classification**	0.408	0.102	16.128	<0.001	1.504	1.233	1.836
**N classification**	0.051	0.121	0.177	0.674	1.052	0.83	1.334
**M classification**	2.267	0.26	75.75	<0.001	9.65	5.792	16.079
**Pathologic differentiation**	1.076	0.498	4.666	0.031	2.933	1.105	7.787

### TCGA data was used to explore the potential functions of GRB7

**Figure 3:**
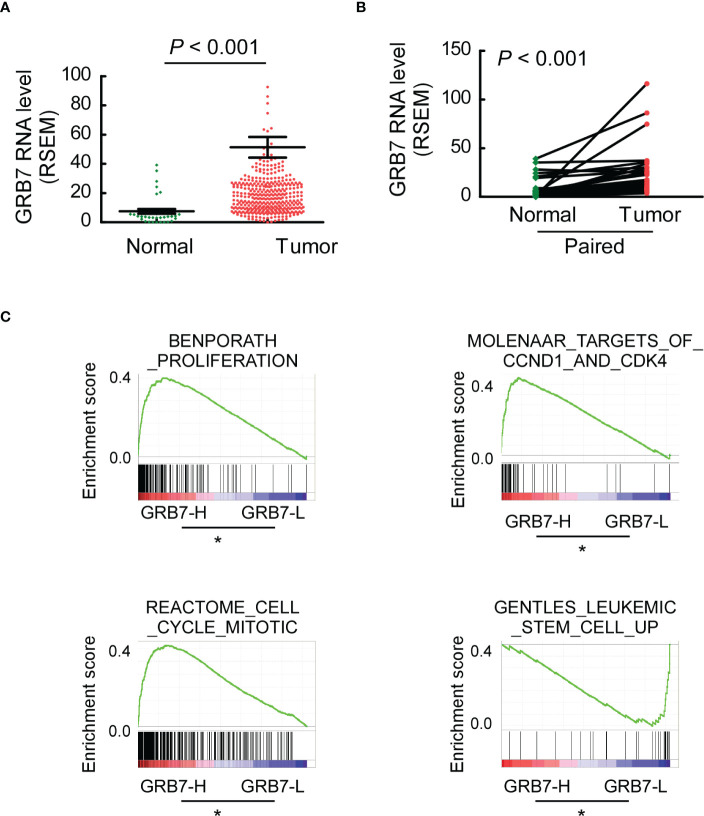
TCGA data indicates the expression trend of GRB7 and the potential correlation with the proliferation and stem cell characteristics. **(A)** GRB7 was significantly higher in 409 cases of gastric cancer than in 37 cases of normal gastric tissue. **(B)** GRB7 in gastric tumor tissue was significantly higher than that in matched tissue from the same patient. **(C)** GSEA analysis showed that GRB7 was positively correlated with the events of accelerating cell growth and increasing stem cells. *P<0.05.

The Cancer Genome Atlas (TCGA) describes a large number of gastric cancer and its matched normal samples at the molecular level. GRB7 was significantly higher in 409 cases of gastric cancer than in 37 cases of normal gastric tissue (**Figure 3A**). GRB7 in gastric tumor tissue was significantly higher than that in matched tissue from the same patient (33 pairs; **Figure 3B**). We first defined the values in TCGA as GRB7 high expression group (GRB7-H)and low expression group(GRB7-L) based on the mean value of GRB7 expression value. Then, Gene Set Enrichment Analysis (GSEA) is used to analyze the signal pathways it may participate in and explore its potential biological functions. GSEA analysis showed that GRB7 was positively correlated with the events of accelerating cell growth and increasing stem cells (**Figure 3C**). Taken together, the TCGA data indicates the expression trend of GRB7 and the potential correlation with the proliferation and stem cell characteristics.

### GRB7 promotes proliferation of gastric cancer cells

**Figure 4:**
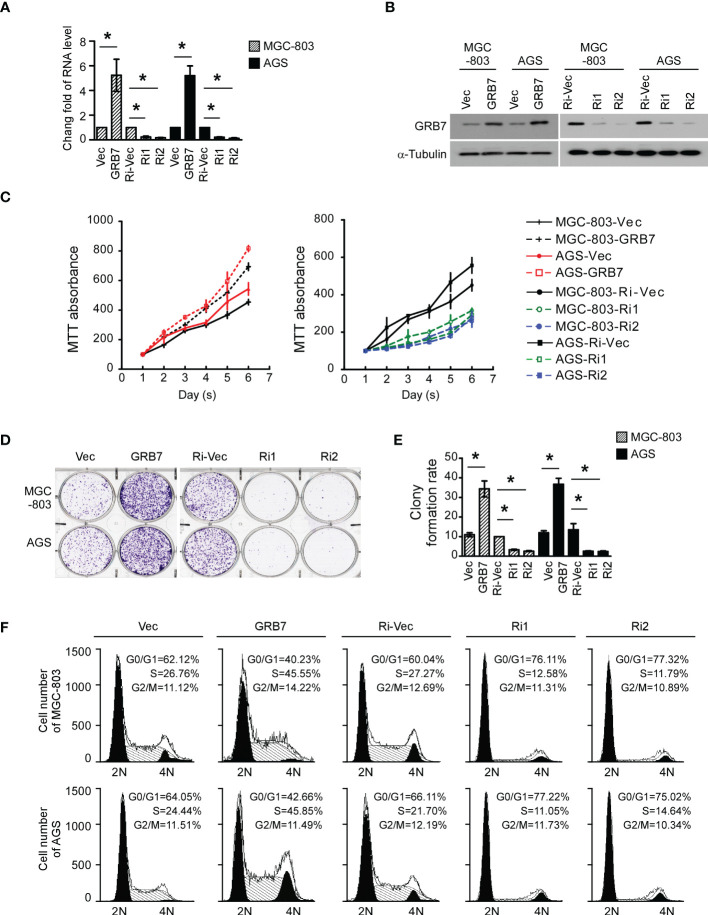
GRB7 promotes proliferation of gastric cancer cells. **(A)** Real-time quantitative PCR analysis showed that gastric cancer cell lines with high/low expression of GRB7 were successfully established. *P<0.05. **(B)** Western blot analysis showed that gastric cancer cell lines with high/low expression of GRB7 were successfully established. **(C)** MTT analysis showed that over-expression of GRB7 significantly accelerated cell proliferation, while low expression of GRB7 slowed down cell proliferation. **(D, E)** The representative pictures and statistical graphs of the colony formation test intuitively show that the expression of GRB7 is positively correlated with the number of colonies (mean ± standard deviation). *P<0.05. **(F)** Flow cytometry analysis described that GRB7 expression is positively correlated with the transition ability of cells in G1-S phase. *P<0.05.

The establishment of stable cell lines with high/low expression of specific genes can be more targeted to study the role of this gene in cells. Real-time quantitative PCR analysis and Western blot analysis showed that gastric cancer cell lines with high/low expression of GRB7 were successfully established(**Figures 4A, B**).

In view of the results of GSEA analysis, we first explored the role of GRB7 in the proliferation of MGC-803 and AGS cells. MTT analysis showed that over-expression of GRB7 significantly accelerated cell proliferation, while low expression of GRB7 slowed down cell proliferation (**Figure 4C**). Representative pictures and statistical graphs of the colony formation test intuitively show that the expression of GRB7 is positively correlated with the number of colonies (**Figures 4D, E**). Flow cytometry analysis is used to detect the proportion of cells in different cell cycle phases in each cell line. As indicated in the figure, when GRB7 was highly expressed, the proportion of cells in the G1 phase decreased by about 20; the proportion of cells in the S phase increased by about 20. On the other hand, when GRB7 expression was low, the proportion of cells in the G1 phase increased by about 15-17; the proportion of cells in the S phase decreased by about 10-15; Under the above two conditions, the proportion of cells in the G2/M phase remained basically unchanged. It can be concluded that GRB7 expression is positively correlated with the transition ability of cells in G1-to-S phase (**Figure 4F**). Taken together, GRB7 promotes proliferation of gastric cancer cells through acceleration of G1/S phase cell cycle transition.

### GRB7 positively correlates with the self-renewal potential of gastric cancer cells

**Figure 5:**
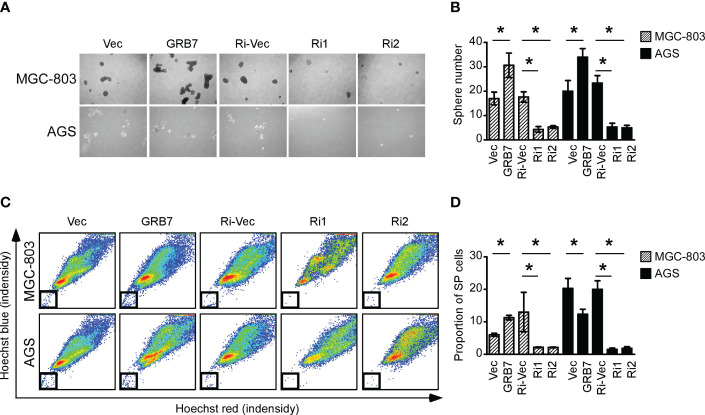
GRB7 positively correlates with the self-renewal potential of gastric cancer cells. **(A**, **B)** The representative pictures and statistical chart of the sphere formation assay showed that the expression of GRB7 is positively correlated with the number of spheres (mean ± SD). *P < 0.05. **(C, D)** Side population stem cell sorting assay revealed that the proportion of SP cells was positively correlated with the expression of GRB7. *P < 0.05.

In view of the results of GSEA analysis, we explored the role of GRB7 in the self-renewal potential of MGC-803 and AGS cells. The spheroidization test is to judge the ability of a single cell to self-renew in a suitable medium, and it can be used to identify the stemness of the cell. The representative pictures and statistical chart of the sphere formation assay showed that the expression of GRB7 is positively correlated with the number of spheres (**Figures 5A, B**). Side population cells are considered to have self-renewal and multidirectional differentiation potential similar to stem cells. Side population sorting assay revealed that the proportion of SP cells was positively correlated with the expression of GRB7 (**Figures 5C, D**). Taken together, GRB7 is essential for the self-renewal phenotype of gastric cancer cells.

## Discussion

GRB7 is a 535 amino acid multi-domain linker protein, which can bind and function with a variety of core proteins in the cell (21). The PH domain of GRB7 has been shown to bind Filamin-A, an actin-binding protein involved in the regulation and control of membrane fold formation (23). The SH2 domain of GRB7 has been shown to bind to various phosphotyrosine motifs of its binding partners, such as EGFR, ERBB2, Ret, platelet-derived growth factor receptor (PDGFR), FAK, etc (24–26). In addition, the interaction between the SH2 domain of GRB7 and the phosphotyrosine motif of the signal protein leads to different cellular outcomes or tumorigenic functions (27). In addition to being initially identified as an EGFR binding partner, GRB7 has also been reported to interact with small GTPases in the Ras superfamily and regulate Ras-mediated cancer progression (28). In summary, GRB7, as the central node connecting multiple potential oncogenic drivers and downstream signaling pathways, must play a huge role in the occurrence and development of tumors. Our current research has found that elevated GRB7 expression is highly correlated with the advanced clinical stage or low survival rate of gastric cancer patients, indicating that GRB7 expression is expected to be a diagnostic and prognostic factor for gastric cancer. It is recognized that GRB7 is related to cell growth. For example, consumption of GRB7 inhibits cell proliferation through the ERK/pathway, promotes apoptosis and reduces tumor xenografts (29); GRB7 is up-regulated during TC development, and regulates TC cell proliferation, cell cycle and mitochondrial apoptosis by activating the MAPK/ERK pathway (30); GRB7 participates in the activation of ERK and Ras by aquaporin-1 to promote the proliferation and invasion of gastric cancer cells (11). GRB7 protein has been reported to play an important role in the transition from G2-M phase of the cell cycle (31). Our current research shows that the GRB7 molecule, which is highly expressed in gastric cancer tissues, can promote the G1-S transition of gastric cancer cells. Additionally, we found that the expression level of GRB7 protein was positively correlated with the self-renewal potential representing tumor cell metastasis, chemotherapy resistance, and recurrence. It can be speculated that treatments targeting GRB7 can fundamentally inhibit the metastasis and recurrence of gastric cancer, which also highlights the important role of GRB7 in tumorigenesis and development.

The cellular localization of GRB7 is closely related to its function. Research has shown that GRB7 protein membrane expression may be associated with a better prognosis in breast and ovarian cancers (14, 15). Deleting the SH2 domain of GRB7 eliminates the subcellular localization of GRB7 in focal contact, thereby inhibiting GRB7-mediated cell migration (32). Studies have found that GRB7 is located in the cytoplasm and nucleus of cells, and acts as a key mediator of the nuclear-cytoplasmic export complex in a signal-dependent manner of EGF (33). Deleting the calmodulin binding domain of GRB7 will ablate its nuclear localization (34). Studies have shown that calmodulin antagonists usually increase the expression of GRB7 in the nucleus while inhibiting cell growth (35). In our present research, we found that GRB7 is mainly located in the cell membrane in normal gastric mucosal tissues, while in malignant cells it is mainly located in the cytoplasm and a small amount is distributed in the nucleus. In summary, we speculate that the cytoplasmic GRB7, not the nuclear GRB7, plays a role in promoting the growth of gastric cancer cells.

GRB7 has become an attractive therapeutic target. For instance, synthetic GRB7-binding peptides, which target to the SH2 domain of GRB7, inhibit the interaction between the GRB7 and ERBB family in breast cancer (36). Chan, D W et al. revealed that targeting GRB7/ERK/FOXM1 signaling pathway impairs aggressiveness of ovarian cancer cells (29). An inhibitory peptide (G7-18NATE) has been developed which binds specifically to the GRB7 SH2 domain and is able to attenuate cancer cell proliferation and migration in various cancer cell lines (37). In addition to direct effects, studies have shown preventing GRB7 accumulation and/or its interaction with receptor tyrosine kinases may increase the benefit of HER2-targeting drugs (38). Our present study indicates that GRB7 molecules highly expressed in gastric cancer tissues can promote the proliferation of gastric cancer cells and increase the proportion of gastric cancer stem cells, so it is expected to become a potential therapeutic target for gastric cancer.

## Data availability statement

The raw data supporting the conclusions of this article will be made available by the authors, without undue reservation.

## Ethics statement

The studies involving human participants were reviewed and approved by Ethics Committee of Longgang District People's Hospital of Shenzhen City and Longgang District Maternity & Child Healthcare Hospital of Shenzhen City. The patients/participants provided their written informed consent to participate in this study.

## Author contributions

YP, LX and FW conduct specimen collection, data sorting and writing. JR and LW are engaged in molecular biology-related experiments. XL conducts cell biology testing. WL is responsible for mining and analyzing network data. All authors contributed to the article and approved the submitted version.
